# The *NtSPL* Gene Family in *Nicotiana tabacum*: Genome-Wide Investigation and Expression Analysis in Response to Cadmium Stress

**DOI:** 10.3390/genes14010183

**Published:** 2023-01-10

**Authors:** Linshen He, Xiang Peng, Hanping Cao, Kunjian Yang, Lien Xiang, Rui Li, Fangyuan Zhang, Wanhong Liu

**Affiliations:** 1 School of Chemistry and Chemical Engineering, Chongqing University of Science and Technology, Chongqing 401331, China; 2 College of Environmental Science & Engineering, China West Normal University, Nanchong 637009, China; 3 School of Life Science, Southwest University, Chongqing 400715, China

**Keywords:** *Nicotiana tabacum* L., *SPL* gene family, cadmium, expression patterns, miR156

## Abstract

The SQUAMOSA promoter binding protein-like (SPL)*SPL* family genes play an important role in regulating plant growth and development, synthesis of secondary metabolites, and resistance to stress. Understanding of the role of the *SPL* family in tobacco is still limited. In this study, 42 *NtSPL* genes were identified from the genome of the tobacco variety TN90. According to the results of the conserved motif and phylogenetic tree, the *NtSPL* genes were divided into eight subgroups, and the genes in the same subgroup showed similar gene structures and conserved domains. The *cis*-acting element analysis of the *NtSPL* promoters showed that the *NtSPL* genes were regulated by plant hormones and stresses. Twenty-eight of the 42 *NtSPL* genes can be targeted by miR156. Transcriptome data and qPCR results indicated that the expression pattern of miR156-targeted *NtSPL* genes was usually tissue specific. The expression level of miR156 in tobacco was induced by Cd stress, and the expression pattern of *NtSPL4a* showed a significant negative correlation with that of miR156. These results suggest that miR156-*NtSPL4a* may mediate the tobacco response to Cd stress. This study lays a foundation for further research on the function of the *NtSPL* gene and provides new insights into the involvement of *NtSPL* genes in the plant response to heavy metal stress.

## 1. Introduction

Cadmium (Cd) pollution of agricultural soil is an increasing problem, and poses a great risk to the growth and development of crops and human health. Reducing the content of heavy metals in edible parts of crops has been a hot topic in the field of plant nutrition and the environment. Tobacco is the most widely cultivated non-food cash crop. Additionally, tobacco has been considered a Cd hyperaccumulator in recent years [1,2]. Therefore, in addition to Cd accumulation in the human body from food and the environment, tobacco is the main source of Cd exposure, especially for smokers [3]. The results of several studies are alarming: they all show that smoking can cause significant Cd accumulation in the body and cause multiple organ dysfunction [4,5]. In addition to active abstinence from smoking, the development of tobacco with low Cd content may be an effective strategy to control the accumulation of Cd in smokers.

The SQUAMOSA promoter binding protein-like (SPL) gene family is a special class of transcription factors in plants. It plays a key regulatory role in plant growth and development and stress resistance [6]. The SPL transcription factors are involved in the regulation of plant growth and development processes, such as tillering and branching in wheat [7], glandular trichome initiation in *Artemisia annua* L. [8], and flowering in cassava [9]. In addition, many studies have shown that SPL transcription factors mediate plant responses to salt stress [10], high temperature [11], low temperature [12,13,14], and other abiotic stresses. In fact, plant SPL transcription factors play a crucial role in maintaining metal ion homeostasis in plants. For example, the miR157-SPL-CNR module negatively regulates the tomato response to iron deficiency [15]. *AtSPL7* is involved in regulating the copper deficiency response of *Arabidopsis thaliana* [16,17]. Recent studies have found that *OsSPL7* regulates the expression of *OsNRAMP5*-mediated Cd accumulation in rice [18]. However, the mechanism of *SPL’s* role in regulating the absorption and transport of metal ions remains unclear in tobacco.

MicroRNAs (miRNAs) are a class of small noncoding RNAs with a length of 18–30 nt that are widely found in plants and animals. On the basis of sequence complement, miRNAs directly act on the mRNA of the target gene and negatively regulate the expression of the target gene through cleavage, inhibition of translation, and DNA methylation [19]. Therefore, miRNAs play an important regulatory role in the growth and development of plants and their response to the external environment [20]. It is important to study the biological functions of miRNAs in regulating the plant response to heavy metal stress to further understand the molecular mechanism of metal metabolism in plants. With the development and wide application of high-throughput sequencing technology, many plant species, including *Sedum alfredii*, *Brassica juncea*, *Medicago truncatula,* and other Cd hyperaccumulators have been studied for miRNAs related to heavy metal responses [21]. The results of such studies suggest that miRNAs are involved in regulating the plant response to heavy metal stress. The results of miRNA transcription data showed that Cd could change miR156 expression patterns in Chinese cabbage, which affects the Cd tolerance of different varieties of cabbage [22]. Overexpression of miR156 in *Arabidopsis* transgenic plants significantly reduces the accumulation of Cd, and enhances Cd stress tolerance in transgenic plants [23]. In fact, there is sufficient evidence that miR156 performs biological functions by regulating *SPL* genes. However, the mechanism by which miR156-*SPL* modulates the plant response to Cd stress has not yet been studied.

Since members of the SPL transcription factor family show pleiotropic functions in plants, genome-wide analysis of the *SPL* gene family has been performed in many plants, including tea plant [24], alfalfa [10,25], *Setaria italica* [26], *Fraxinus mandshurica* [27], *Ziziphus jujuba* [28], *Chenopodium quinoa* [29] and sugarcane [30]. In these studies, the functional diversity of SPL transcription factors was revealed and they played an important role in resisting external stress. However, there have been no reports on the genome-wide identification of the *NtSPL* gene family. In this study, basic information on *NtSPL* gene family members was extracted from the genome of tobacco variety TN90, and phylogenetic tree analysis, gene structure analysis, promoter *cis*-acting element prediction, and targeting relationship prediction between miR156 and *NtSPL* were performed. Finally, qPCR was used to assay the tissue expression patterns of some *NtSPL* genes and their expression patterns under Cd stress. These results provide an important basis for revealing the regulation of the miR156-*NtSPL* module on Cd uptake and accumulation in tobacco.

## 2. Materials and Methods

### 2.1. Identification and Sequence Analysis of the NtSPL and Nta-miR156 Gene Family

The completed whole genome data of the tobacco cultivar TN90, including amino acid sequences and functional annotations of all the proteins were downloaded from the NCBI database (https://www.ncbi.nlm.nih.gov/, accessed on 12 January 2020). The HMM profile of SBP (PF03110) was downloaded from the Pfam database (http://pfam.xfam.org/, accessed on 12 October 2020) and the HMMER program (http://hmmer.org, accessed on 12 October 2020) was employed to filter out the tobacco NtSPL protein sequence. The conserved SBP domain of candidate NtSPL proteins was verified with the Pfam database (http://pfam.xfam.org/, accessed on 21 November 2020) and Smart database (http://smart.embl.de/smart/batch.pl, accessed on 21 November 2020), and redundant protein sequences were removed. Finally, 42 *NtSPL* genes were accurately screened. The relative molecular weights and theoretical isoelectric points of tobacco NtSPL proteins were calculated using the ProtParam tool in ExPASyweb (http://www.expasy.org/, accessed on 16 April 2021), while the subcellular localization of NtSPL proteins was predicted using the Plant-mPLoc online website (http://www.csbio.sjtu.edu.cn/bioinf/plant-multi/, accessed on 10 August 2022) [31].

Sequence information of miR156 family members from a total of six species of *Arabidopsis*, rice, maize, tomato, oilseed rape, and tobacco was downloaded from the PmiREN 2.0 database (https://pmiren.com/, accessed on 17 February 2022) [32]. Multiple sequence alignment of Nta-miR156 matrices was performed using the ClustalW program of MEGA 11 software, and default parameters were selected for the alignment process. Subsequently, the results of the multiple sequence comparison were visualized by Jalview 2.11 software. Using the same method, the mature sequences of miR156 family members were aligned in five model plants (*Arabidopsis*, rice, maize, tomato, and oilseed rape), and the miR156 sequence Logo of these model plants and the tobacco miR156 sequence Logo was mapped by the WebLogo 2.8 online tool (https://weblogo.berkeley.edu/logo.cgi, accessed on 5 March 2022) [33].

### 2.2. Phylogenetic Analysis

The 42 tobacco NtSPL proteins, 17 *Arabidopsis* AtSPL proteins [34], 15 tomato SlySPL proteins [35], and 22 *M. truncatula* MtSPL proteins [36] were compared by MUSCLE multiple sequence alignments using MEGA 11 software, and a maximum likelihood (ML) phylogenetic tree was then constructed using the LG protein model with other parameters set to default. The classification of the tobacco *NtSPL* gene family was based on the classification method of the tomato *SlySPL* gene family. Finally, the phylogenetic tree was visualized using the EvolView online website (https://evolgenius.info//evolview-v2/#login, accessed on 3 January 2023) [37].

### 2.3. Multiple Sequence Alignment Analysis and Motif Composition and Gene Structure

Multiple sequence alignment of the *NtSPL* gene family was performed using the MUSCLE algorithm in JalView 2.11 software [38], followed by visual analysis of the multiple sequence alignment results using JalView 2.11 software, which was used to construct a consistent sequence of *NtSPL* family members [39]. The online MEME tool (http://meme-suite, accessed on 14 November 2022) was used to determine the distribution of conserved motifs in NtSPL proteins by setting 12 motifs, with an unlimited number of motif occurrences on each sequence and default values for all other parameters [40]. The online tool GSDS (http://gsds.gao-lab.org/, accessed on 14 November 2022) was used to generate *NtSPL* gene structure maps based on the available tobacco coding sequences and their respective genomic sequences [41]. Finally, the visualizations of the phylogenetic tree, conserved motif distribution, and gene structure of the tobacco *NtSPL* gene was constructed using the Gene Structure Viewer program in Tbtools 1.108 [42].

### 2.4. Prediction of Cis-Acting Elements

The 1500 bp sequence upstream of the CDS of the *NtSPL* gene was extracted as the promoter sequence using the Gtf/Gff3 Sequences Extract program in the Tbtools 1.108 tool, and the extracted promoter sequence was submitted to the PlantCARE online website (http://bioinformatics.psb.ugent.be/webtools/plantcare/html/, accessed on 6 May 2021) for *cis*-acting element prediction analysis [43]. Finally, the screened tobacco *NtSPL cis*-acting elements were visualized using the Simple BioSequence Viewer program in TBtools 1.108.

### 2.5. Predictive Analysis of NtSPL Gene Family miRNAs

To better understand the miRNAs regulating *NtSPL* gene expression, we submitted the mRNA sequences with *NtSPL* genes to the psRNATarget online server (http://plantgrn.noble.org/psRNATarget/, accessed on 13 May 2022). The expectation was adjusted to 1, and other parameters were predicted by default analysis [44]. Subsequently, Venn and Sankey plots were drawn to demonstrate the distribution relationship between Nta-miR156 and the *NtSPL* gene family through the mapping tool provided by the SangerBox online platform (http://vip.sangerbox.com/, accessed on 26 May 2022) [45]. To further understand the mode of action between Nta-miR156 and its target genes, the sequence logos of all target genes and miR156 interaction regions were mapped by the WebLogo 2.8 online tool, and then the target binding pattern between Nta-miR156 and the *NtSPL* gene family was mapped by combining the sequence logos of tobacco miR156.

### 2.6. NtSPL Expression Patterns by Transcriptome Data

To investigate the expression patterns of the *NtSPL* gene family in different tissues of tobacco, the raw transcriptome data (PRJNA208209) was downloaded from the NCBI database (http://www.ncbi.nlm.nih.gov/, accessed on 28 September 2020) for expression analysis of TN90 tobacco, including raw transcriptome data for root, stem, young leaf, mature leaf, senescing leaf, young flower, and mature flower and SRA data for senescing flower [46]. 

All RNA-seq data were first quality assessed using FastQC, followed by quality control cleaning, and the relative expression level TPM values of the *NtSPL* gene family were obtained by further computational analysis of the transcriptome data based on tobacco genomic data through Kallisto 0.46.2 software for gene expression [47]. Finally, the HeatMap tool of TBtools 1.108 was used for visualization.

### 2.7. Plant Materials and Heavy Metal Treatments

Seeds of tobacco variety TN90 were sown in uncontaminated nutrient soil and incubated at 25 ± 2 °C with 65–75% relative air humidity, 2000 lux light intensity, and a 16/8 h light/dark cycle. Two-week-old tobacco seedlings were transplanted into 1/2 Hoagland solution for 7 days. The solution in the hydroponic tank was then replaced with a fresh Hoagland solution containing 50 μM CdCl_2_. Tobacco seedlings were incubated with the above treatments for 7 days. The leaves and roots of the tobacco seedlings were then harvested in stages. Plant samples were snap-frozen in liquid nitrogen and stored in a −80 °C refrigerator for subsequent plant RNA extraction.

To analyze tissue expression profiles, we collected five tissue samples, including roots, stems, old leaves, young leaves, and flowers, from 4-month-old tobacco plants grown in their natural environment. All samples were immediately frozen in liquid nitrogen and stored at −80 °C until total RNA was isolated. Each group included three biological replicates and technical replicates.

### 2.8. Analysis of the Pattern of NtSPL Gene Expression

RNA was extracted from the above plant material according to the instructions of the RNAsimple Total RNA Extraction Kit (Catalog No. DP419 and DP424) from Tiangen, Beijing, China. cDNA was synthesized from the first strand of the plant samples according to the GoScript^TM^ Reverse Transcriptase Kit from Promega, Madison, USA, and the synthesized cDNA was used for subsequent qPCR analysis. Three tobacco seedlings were used in each group for RNA extraction experiments.

qPCR was performed on the CFX96^TM^ real-time fluorescence quantification platform using SYBR green luciferase (Novoprotein, Suzhou, China) with a reaction program of 45 cycles of 95 °C for 1 min; 95 °C for 15 s; and 60 °C for 30 s. The qPCR primers for the Nta-miR156 and *NtSPL* gene family were designed by Primer Premier 6.0 (Table S1). Tobacco *NtEF1α* (accession number: AF120093) was used as an internal reference gene [48], and the tissue expression profile of the *NtSPL* gene family and the relative expression of the *NtSPL* gene family under Cd stress were calculated using the 2^−ΔΔCT^ method [49].

## 3. Results

### 3.1. Identification and Phylogenetic Analysis of NtSPL Genes

According to the HMM search results, 42 candidate genes were obtained from the tobacco *NtSPL* gene family. Among them, 40 *NtSPL* genes have a completely conserved SBP structural domain (PF03110), and interestingly, the two genes *NtSPL2b* and *NtSPL4g* lack the complete SBP domain.

**Figure 1:**
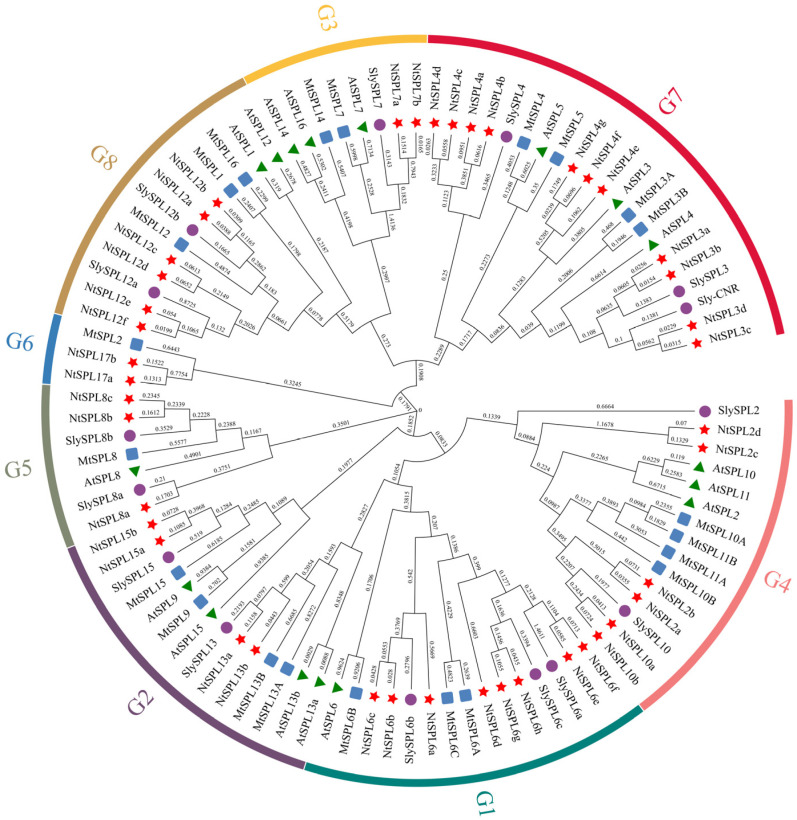
Phylogenetic analysis of SPL proteins from *Arabidopsis*, *M. truncatula*, *Solanum lycopersicum,* and *Nicotiana tabacum*. The maximum likelihood (ML) phylogenetic tree was constructed using full-length SPL protein sequences in MEGA 11. *AtSPL* is represented by green triangles, *MtSPL* by blue squares, *SlySPL* by purple circles, and *NtSPL* by red stars.

To further understand the evolutionary relationships between *NtSPL* genes and *SPL* genes in other species, we constructed ML phylogenetic developmental trees using SPL protein sequences from *Arabidopsis*, tobacco, and tomato (Figure 1) to further elucidate the evolutionary relationships of *SPL* genes in tobacco. All *NtSPL* genes were assigned specific names based on the phylogenetic relationships with their more closely related tomato *SlySPL* genes. The results of the phylogenetic analysis showed that the 96 SPL proteins were clustered into eight different taxa (named G1–G8) (Figure 1). Among them, the large subclade G7 contained 11 *NtSPL* genes, which were mainly divided into two subclade members, *NtSPL3* and *NtSPL4*. Notably, tobacco *NtSPL17a* and *NtSPL17b* are separately clustered as the G6 large subclade, and may be the *NtSPL* genes specific to the evolutionary development of tobacco. 

In this study, we characterized the protein sequences and physicochemical properties of NtSPL family members. The range of the *NtSPL* gene CDS lengths was 357~3003 bp, and the range of the encoded NtSPL protein lengths was 119~1001 amino acids. The range of the NtSPL protein molecular weights was 13.7~111.3 kDa, and the range of the theoretical isoelectric point (pI) of the proteins was 5.09~9.65. In addition, the predicted subcellular localization results showed that all *NtSPL* genes were localized in the nucleus, with six genes, *NtSPL2a*, *NtSPL2c*, *NtSPL7a*, *NtSPL7b*, *NtSPL10a,* and *NtSPL10b*, predicted to be localized not only in the nucleus but also in the cytoplasm (Table S2).

### 3.2. Multiple Sequence Alignment of NtSPL Proteins

**Figure 2:**
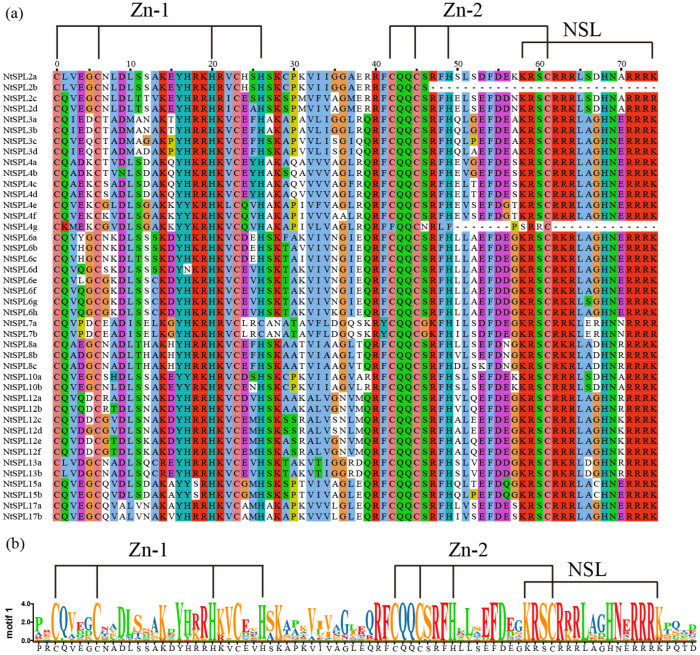
Multiple sequence alignment of SBP structural domains of the *NtSPL* gene family. (**a**) Multiple alignments of SBP structural domains of NtSPL proteins using Jalview software showing two conserved zinc finger structures (Zn-1, Zn-2) and an NLS. (**b**) Motif logo and protein sequence of the SBP domain and NLS segment, and the height of letters in each pair stack represents the relative frequency of the corresponding amino acids.

The differences between the 42 NtSPL proteins were analyzed using multiple sequence alignment, and the results of multiple sequence alignment (Figure 2) showed that all NtSPL transcription factor family member except NtSPL2b and NtSPL4g contains a highly conserved SBP domain consisting of 79 amino acid residues. They all contain two zinc finger structures Cys-Cys-His-His (C2H2), and Cys-Cys-His-Cys (C3H), and a nuclear localization signal (NLS). Among the NtSPL proteins, NtSPL7a and NtSPL7b have an N-terminal zinc finger structure of Cys4 (C4), which is different from the other tobacco NtSPL members. Notably, the lack of the NLS nuclear localization signal in the SBP domains of the *NtSPL2b* and *NtSPL4g* genes may prevent them from performing normal physiological functions. 

### 3.3. Conserved Motifs and Gene Structure Analysis of NtSPL Genes

**Figure 3:**
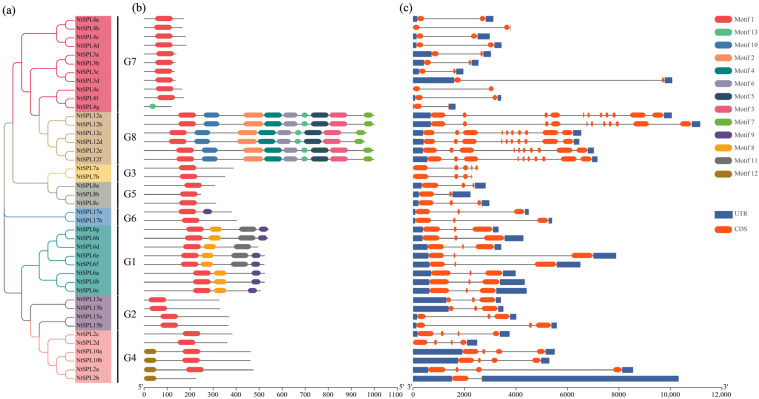
Conserved motifs and gene structures of the *NtSPL* gene family. (**a**) The maximum likelihood (ML) phylogenetic tree of 42 NtSPL proteins was constructed using MEGA 11; (**b**) Distribution of conserved motifs in NtSPL proteins. Different motifs are represented by the different colored boxes; (**c**) Exon-intron structure of the *NtSPL* gene. Lines represent introns, blue boxes represent UTRs, and red boxes represent exons.

A phylogenetic tree was constructed based on the protein sequence of NtSPL, and the gene family was divided into eight groups. Group G7 was the largest with 11 members, but groups G3 and G6 had only two NtSPL members (Figure 3a). We further analyzed the conserved motifs of *NtSPL* family genes and identified 12 motifs using MEME software with default parameters (Figure 3b). As expected, all NtSPL proteins except NtSPL2b and NtSPL4g contain Motif1, the SBP structural domain, consisting of approximately 79 amino acids. Notably, except for the G1 and G8 subfamilies, the *NtSPL* genes contain only Motif1, which is relatively more conserved. Most closely related members of the same subfamily have a common motif composition, and members in the G1 and G8 subfamilies possess some structural domains that are absent or atypical in other subfamilies. The differential distribution of these conserved motifs may be responsible for the differences in gene function.

The evolution of the tobacco *NtSPL* gene family was further explored by studying the intron-exon structure of tobacco *NtSPL* genes. The results showed that the number of intron structures in the *NtSPL* gene family ranged from 1 to 10 (Figure 3c). The G8 subfamily had the highest number of introns, with an average of 9.3 introns; the G7 subfamily contained only one intron; the G1, G2, G5 and G6 subfamilies contained only two introns; and the G3 and G4 subfamilies contained intron numbers ranging from 1 to 4.

### 3.4. Cis-Acting Elements in the Promoter Regions of NtSPL Genes

**Figure 4:**
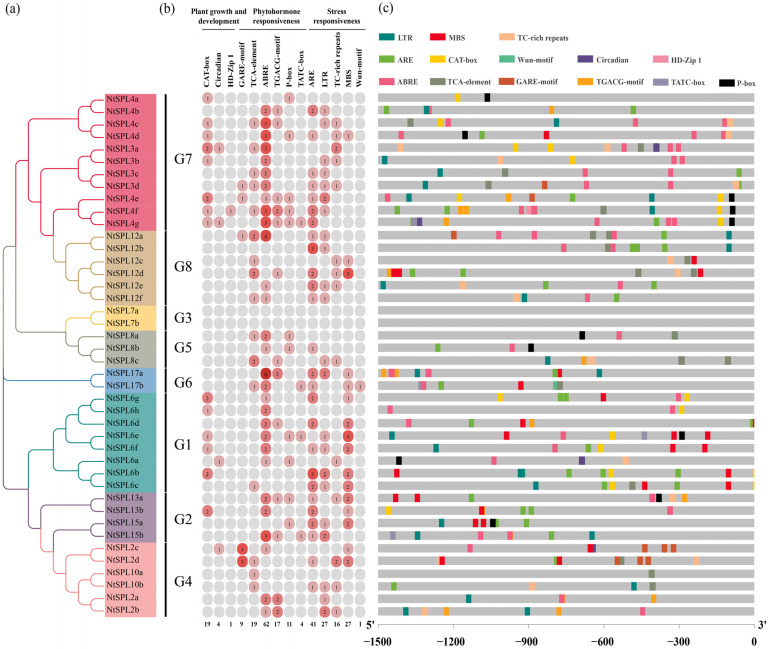
*Cis*-acting elements of the *NtSPL* gene family. (**a**) The maximum likelihood (ML) phylogenetic tree of 42 NtSPL proteins was constructed using MEGA 11; (**b**) Frequencies of *NtSPL cis*-acting elements are indicated by numbers and shaded in different colors; (**c**) Distribution of *NtSPL* cis-acting elements on promoters.

To better understand the potential regulatory mechanisms of *NtSPL* genes in tobacco in regard to abiotic stress responses, phytohormone responses, and growth and development, we further analyzed the upstream 1.5 kb promoter of the tobacco *NtSPL* genes. Fourteen elements involved in plant growth and development, plant hormone response, plant defense, and stress-related elements were screened in the *NtSPL* promoter and classified into three categories. These elements are irregularly scattered in the promoter regions of *NtSPL* family genes and are unique (Figure 4 and Table S3). 

A variety of hormone-related *cis*-acting elements were identified in the promoter region of the *NtSPL* gene, including the GARE-motif/P-box/TATC-box element associated with gibberellin, the TCA-element element associated with salicylic acid, the ABRE associated with abscisic acid and the TGACG-motif element associated with MeJA. Among them, ABREs and TCA elements were widely distributed in the *NtSPL* promoter sequence, indicating that abscisic acid and salicylic acid are widely involved in the regulation of *NtSPL* genes. These results suggest that phytohormones may play an important role in the regulation of *NtSPL* gene expression.

The plant meristem-associated component CAT-box is mainly found in the tobacco *NtSPL3*, *NtSPL4*, and *NtSPL6* subfamilies. The *cis*-regulatory element circadian, which is involved in the regulation of plant circadian rhythms, is present in a small number of the *NtSPL* genes in tobacco, namely, *NtSPL3a*, *NtSPL4g*, *NtSPL6a*, and *NtSPL2c*. Similarly, the HD-Zip1 element involved in plant leaf cell differentiation was present only in *NtSPL4f*. These results suggest that *NtSPL* gene expression plays an important role in plant growth and development.

The anaerobic-inducible regulatory-associated element (ARE) is also widely present in the promoters of *NtSPL* family genes, especially in the promoter regions of *NtSPL6b* and *NtSPL12b*, which contain three AREs. In addition, among environmental stress-related factors, the low-temperature response element LTR, the drought-inducible element MBS, and TC-rich repeats, elements involved in plant defense and stress responses, were widely distributed in *NtSPL* genes. Interestingly, the response plant wound healing element Wun-motif was present only in *NtSPL17b*. These results suggest that the expression of *NtSPL* genes may be correlated with external environmental factors. Notably, the above three related *cis*-acting elements were not found in the promoter of *NtSPL7a* or *NtSPL7b*, indicating the possible existence of other different regulatory pathways.

### 3.5. Nta-miR156 Multiplex Sequence Alignment

The miR156 family in tobacco is a superfamily containing 20 members with nomenclature ranging from Nta-miR156a to Nta-miR156t. The multiple sequence alignment results showed that the mature sequences of tobacco miR156 family members are highly similar, and some sequences are even identical. The results showed that the entire tobacco miR156 family has only 20 bases for the three mature sequences Nta-miR156f, Nta-miR156r, and Nta-miR156l, 22 bases for Nta-miR156e and Nta-miR156h, and 21 bases for the rest of the miR156 members (Figure S1). In tobacco, mature miR156 was classified into five groups based on core sequences at positions 2–21. Group I has 14 members with a mature core sequence of 5′-UGACAGAAGAGAGAGUGAGCAC-3′. Group II has three members, Nta-miR156i, Nta-miR156p, and Nta-miR156q, with a mature core sequence of 5′-UGACAGAAGAGAGAGAGAGCAC-3′. Groups III, IV, and V each have one member, Nta-miR156f, Nta-miR156r, and Nta miR156l, respectively, and their core sequences differ from those of all other miR156 members. 

To further understand the differences between miR156 in tobacco and other species, the sequence conservation patterns of Nta-miR156 (20 members) and the five model species (63 members) were mapped by the WebLogo tool. These model species included *Arabidopsis* (10 members), maize (13 members), rice (13 members), oilseed rape (20 members), and tomato (7 members). Based on the analysis, the core sequence 5′-UGACAGAAGAGAGUGAGCAC-3′ at bases 2–21 of the tobacco miR156 family and other model species miR156 families is highly conserved (Figure S1).

### 3.6. Analysis of miR156 and Its Target Sequences

Many studies have shown that most *SPL* genes are regulated by miR156 and that miR156 target sites are located in the coding region or 3′UTR. To investigate the posttranscriptional regulatory mechanism of tobacco *NtSPL*, we predicted the targeting relationship of Nta-miR156 and *NtSPL* online through the psRNATarget online server. The results showed that 28 *NtSPL* genes were potential targets of miR156, and these genes were mainly distributed in the G1, G2, G4, G6, and G7 subgroups. *NtSPL* with potential targets of miR156 in the coding region belonged to the G1, G2, G4, and G6 subgroups, while those with targets in the 3′UTR belonged to the G7 subgroup (Figure S2).

**Figure 5:**
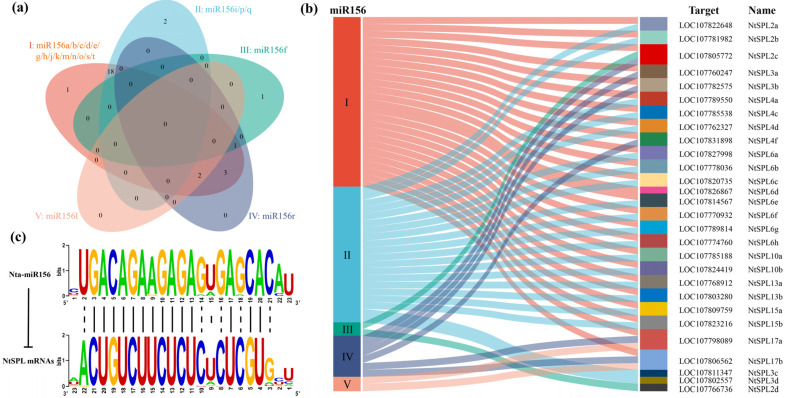
Target gene prediction for tobacco Nta-miR156. (**a**) Venn diagram of the Nta-miR156 targeting relationships for the five groups. (**b**) Sankey diagram of the distribution of the five groups of Nta-miR156 with *NtSPL* target genes. (**c**) Target binding relationship between Nta-miR156 and *NtSPL*. The upper sequence pattern is the sequence Logo of 20 Nta-miR156 mature sequences, and the lower sequence pattern is the sequence Logo of *NtSPL* target genes with Nta-miR156 targeting regions. The solid line in the middle indicates a highly conserved base linkage in both patterns, while the dashed line indicates slightly less conserved base linkage.

Since the core of the targeting relationship is the complementary pairing of bases, the targeting relationship of the five groups of Nta-miR156 is demonstrated by a Venn diagram (Figure 5a). Group I Nta-miR156 has a total of 25 target genes, of which only one is unique to that group and 18 are shared with Nta-miR156i/p/q (Group II). The other six target genes of Group I were common to Nta-miR156r (Group IV), which also shared one target gene with Nta-miR156f (Group III) and two target genes with Nta-miR156l (Group V). 

The Sankey plot clearly demonstrates the distribution between the five Nta-miR156 groups and their target genes (Figure 5b), with Group I and Group II targeting most of the *NtSPL* target genes. In the binding relationship between Nta-miR156 and the target gene *NtSPL* (Figure 5c), Nta-miR156 is bound very tightly to the mRNA of the *NtSPL* gene. Bases 1, 22, and 23 of the Nta-miR156 sequence are rarely involved in target binding to the *NtSPL* gene, while bases at position 15 and adjacent positions may be critical for differences in targeting relationships. 

### 3.7. Expression Pattern Analysis Based on Transcriptome Data

The results of *NtSPL* gene expression in eight different tissues showed that *NtSPL12a*, *NtSPL12b*, *NtSPL12d*, *NtSPL12e*, *NtSPL12f*, and *NtSPL7a* were highly expressed in all tissues of tobacco. Interestingly, the tobacco *NtSPL4a* gene was barely expressed in the roots, however, it was highly expressed in all other tissues. Among the *NtSPL* genes, *NtSPL2c*, *NtSPL2d*, *NtSPL4b*, *NtSPL4e*, *NtSPL4g*, *NtSPL8c*, *NtSPL17a*, and *NtSPL17b* had a tissue-specific expression, and all of them were expressed at relatively low levels (Figure S3).

### 3.8. Tissue-Specific Expression Profiles of NtSPL Family Genes

**Figure 6:**
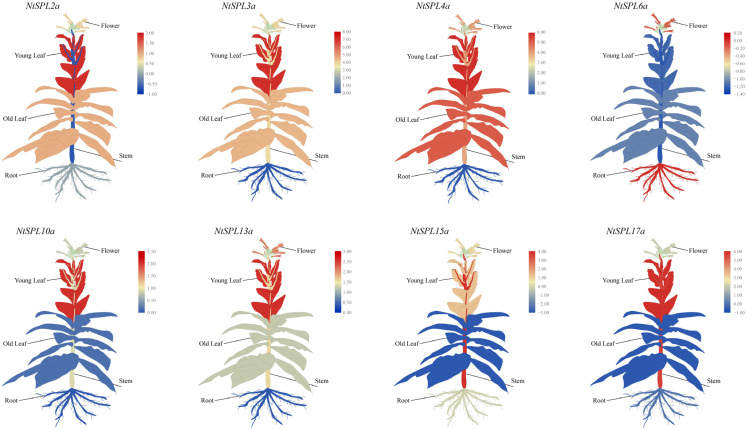
The relative expression levels of *NtSPL* genes in different tobacco tissues. The expression data were obtained from the real-time RT-PCR (qPCR) analysis and are shown as log2 values calculated as averages. The expression level of *NtSPL* in the root is defined as 1 (log2 = 0). Data are presented as the means ± SD of three replicates. High expression levels are shown in red, and lower expression levels are shown in blue.

To reveal the tissue expression patterns of the *NtSPL* family genes, tissue samples of tobacco roots, stems, old leaves, young leaves, and flowers were collected in this study, and the relative expression levels of the *NtSPL* family genes in the five tobacco tissues types were quantified using qPCR. The expression results showed that *NtSPL2a*, *NtSPL3a*, *NtSPL4a*, *NtSPL10a*, *NtSPL13a*, and *NtSPL17a* were all highly expressed in the young leaves, and *NtSPL2a*, *NtSPL3a*, *NtSPL4a*, and *NtSPL13a* were also highly expressed in the old leaves (Figure 6). It was clear that all eight *NtSPL* genes had relatively high expression in tobacco flowers. Interestingly, only *NtSPL6a* was expressed at a relatively high level in roots, while the remaining seven *NtSPL* genes were expressed at a relatively low level in roots. In addition, all *NtSPL* genes except *NtSPL2a* and *NtSPL6a* were relatively highly expressed in tobacco stems, with *NtSPL15a* and *NtSPL17a* having the highest relative expression in stems. 

### 3.9. Heavy Metal Stress-Induced Expression Profiles of NtSPL Family Genes

**Figure 7:**
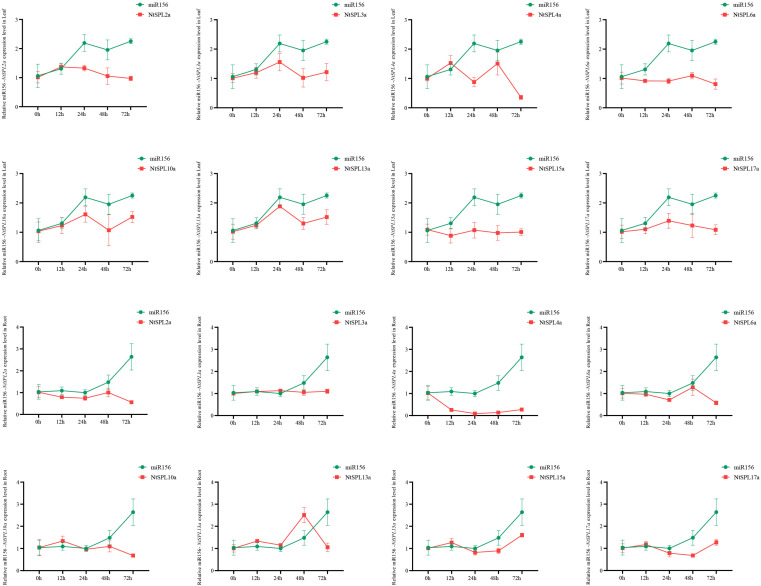
Relative expression levels of Nta-miR156 and *NtSPL* gene family in tobacco Roots and Leaves under Cd stress. Data are presented as the means ± SD of three replicates.

To analyze the relationship between the expression of Nta-miR156 and the *NtSPL* family genes in tobacco under external heavy metal Cd stress, the relative expression data of Nta-miR156 and *NtSPL* under exposure to 50 μM Cd^2+^ stress for different times were determined. The results showed that the expression of Nta-miR156 was significantly upregulated in tobacco in both aboveground parts and roots under 1 to 3 days of Cd stress (Figure 7). Interestingly, under heavy metal Cd stress, only the expression level of tobacco *NtSPL4a* was negatively correlated with the expression level of Nta-miR156. The results suggest that Nta-miR156 may further maintain plant endostasis by regulating the gene expression level of *NtSPL4a* in response to heavy metal Cd stress. 

## 4. Discussion

SPL transcription factors regulate morphogenesis [50,51], growth and development [52], secondary metabolite biosynthesis [53,54], and Cu homeostasis [55] in plants. Additionally, previous studies have revealed that *SPL* genes are involved in mediating plant responses to abiotic stresses [56]. To date, genome-wide identification and characterization of the *SPL* gene family have been documented in crops, including rice, grape, and citrus [34,57,58]. However, genome-wide analysis of the *SPL* gene family remains unclear in *N. tabacum*. In the present study, 42 *NtSPL* genes were identified from the tobacco cultivar TN90, and the evolutionary relationship, gene structure, *cis*-acting elements, and miR156 target gene prediction of *NtSPL* family genes were comprehensively analyzed. Additionally, qPCR was used to analyze the expression levels of *NtSPL* genes in different tissues and in tobacco under Cd stress.

SPL family is a family of plant-specific transcription factors that exist in both *Chlamydomonas reinhardtii* and higher plants [59]. It is generally assumed that plant *SPL* family genes originate from green algae, and the *SPL* family genes of land plants are divided into two classes: Class I SPL proteins are characterized by four conserved Cys residues in the zinc finger structure of the N-terminus, while in Class II SPL proteins, the fourth Cys is replaced with His [60]. Based on the *SPL* family gene sequences of *A. thaliana* and tomato, the 42 members of the tobacco *NtSPL* gene family were clustered into eight branches in the evolutionary tree (Figure 1). The G3 subgroup contains four members, *NtSPL7a*/*7b*, *AtSPL7,* and *SlySPL7*, all of which belong to Class I SPL proteins due to the C4 conserved motifs (Figure 2). The other seven subfamily members in tobacco were classified as Class II SPL proteins. The phylogenetic tree was consistent with the results in *C. quinoa*, *Fagopyrum tataricum*, and *Malus* × *domestica* Borkh [29,61,62], which suggested a similar convergent evolutionary pattern of the *SPL* family in plants.

The functional diversity of *SPL* family genes may be related to the differences in gene structure in plants. There are significant differences in exon-intron patterns among members of the *NtSPL* gene family in the tobacco plant (Figure 3), which may be caused by intron and exon loss after gene replication [60]. In addition, the motif composition of *NtSPL* was similar in the same branch of the phylogenetic tree, which indicated that duplicated *SPL* gene pairs exist in tobacco as in other species [63]. However, there were differences in motif composition in different branches of the tobacco *NtSPL* gene family, suggesting that these genes perform different biological functions. Although the functions of most *SPL* genes have been reported to be related to the regulation of plant growth and development, some *SPL* genes that maintain metal homeostasis are noteworthy in plants. The *AtSPL7* gene in the G3 group binds to the GTAC motif (Cu-responsive element) in the miR398 promoter to regulate Cu homeostasis in *A. thaliana* [17,64]. The *Sly-CNR* gene in the G8 group negatively mediates the iron deficiency response by regulating the expression of the Fe homeostasis-related transcription factor bHLH101 in tomato [15]. In addition, *AtSPL7* showed a response to Cd stress [65]; thus, this protein may be independently involved in the regulation of Cd tolerance and the accumulation of miR156 [23]. Therefore, the study of the regulation of metal homeostasis by G3 and G8 subfamily members of *NtSPL* family genes in tobacco is worth investigating in the future.

The *cis*-acting element analysis revealed that *NtSPL* family genes were widely involved in a variety of physiological processes, including plant growth and development, plant hormone responses, and plant stress responses. We analyzed 14 *cis*-acting elements in the promoter regions of *NtSPL* family genes and found that the promoters of fourteen *NtSPL* genes contained a CAT box, which is a regulatory element associated with meristem expression. SPL transcription factors mediate the establishment of meristem boundaries in plants [66]. Among all the regulatory elements related to the hormone response, ABREs related to the ABA response were the most abundant in the *NtSPL* gene promoter region, which is consistent with the results in other species including tea plant and apple [62,67]. A recent study reported that SPL directly activated the expression of ABA-responsive genes through interaction with abscisic acid-sensitive 5 (ABI5) [68]. The P-box, a regulatory element responding to gibberellin, is widely present in the promoter region of *NtSPL* genes (Figure 4). There has been evidence that the GA signaling pathway protein DELLA mediates axillary meristem (AM) growth and controls collateral formation by regulating *SPL9* expression [52]. Anaerobic inducible associated elements (AREs) are the most prevalent *cis*-acting elements associated with the stress response in the promoter region of *NtSPL* genes (Figure 4). The SBP domain gene *Crr1* is involved in inducing gene expression in response to anaerobic stress in *C. reinhardtii* [69]. In addition, several stress response-related elements including LTRs, TC-rich repeats, and MBSs, exist widely in *NtSPL* gene promoter regions, suggesting that *NtSPL* plays an important role in mediating the plant response to abiotic stress.

It is well known that the miR156-*SPL* module is the regulatory center mediating various physiological processes in plants. In this study, 20 sequences of miR156 genes were isolated from the tobacco database, and divided into five groups based on the consistency of seed sequences (Figure S1). All miR156 miRNAs in Group I, which has the most members, contained the core seed sequence 5′-UGACAGAAGAGAGUGAGCAC-3′, while the mutation site in other groups was mainly located at the 15th base at the 5′-end of the miR156 seed sequence (Figure S1). This result was similar to previous studies [70]. In addition, only 28 of the 42 *NtSPL* genes contained the target site of miR156 (Figure 5). In *A. thaliana*, only 10 of the 17 *SPL* genes were targeted by miR156, and 7 of the 17 *SPL* genes in barley contained complementary sequences for miR156 [71]. The target site of miR156 is in the coding region of the *NtSPL* gene or 3′-UTR in tobacco (Figure S2), which is consistent with the results in *M. truncatula* and *Z. jujuba* [28,36]. These results indicate that the miR156 family of tobacco, similar to other species, maintains a high degree of conservation.

The *NtSPL* family genes showed different expression patterns in different tissues of tobacco plants. Based on the transcriptome data, the *NtSPL* family genes were classified into three types, including the constitutional high expression level type, tissue-specific expression type, and constitutional low expression level type (Figure S3). Most members of the *NtSPL12* subfamily were constitutionally expressed and maintained relatively high expression levels (Figure 5), most likely because these genes are not targeted by miR156 [72]. The expression level of *NtSPL2c* remained low in tobacco tissues except in senscent flower, because the predicted results showed that three groups of miR156 genes targeted the *NtSPL2c* gene. Meanwhile, eight predicted miR156-targeted *NtSPL* genes were verified by qPCR, and the results showed that the expression levels of the eight genes were generally consistent with the results of the transcriptome data (Figure 6). These results confirm that miR156 plays a key role in the regulation of the miR156-targeted *NtSPL* gene in plant growth and development.

miR156 has been confirmed to regulate Cd accumulation and enhance Cd tolerance in transgenic *A. thaliana* plants [23]. In addition, a previous study showed that the expression levels of *SPL* genes could be induced in plants under Cd stress [29]. However, there is still no evidence that the miR156-*SPL* module mediates plant responses to Cd stress. Therefore, eight miR156-targeted *NtSPL* genes and miR156 expression patterns were investigated in tobacco plants under Cd stress in this study. The expression level of miR156 was significantly upregulated in the roots and shoots of tobacco plants under Cd stress (Figure 7). However, the expression patterns of eight putative miR156-targeted *NtSPL* genes were different. Among them, the expression pattern of *NtSPL4a* in tobacco showed a high negative correlation with the expression pattern of Nta-miR156 (Figure 7), suggesting that the mi156-*NtSPL4a* module is the key module for the tobacco plant response to Cd stress. These results provided a new candidate gene for the study of mi156 in mediating the plant response to Cd stress.

## 5. Conclusions

In this study, 42 *SPL* genes containing the SBP domain were identified in the tobacco variety TN90. The *NtSPL* gene family was divided into eight branches, and members of the same branch had similar domain compositions and intron-exon structures. The 20 members of Nta-miR156 from tobacco could be classified into five groups, and 28 of the 42 *NtSPL* genes were miR156-targeted genes. According to transcriptome data, the expression patterns of *NtSPL* family genes in different tissues of tobacco plants could be clustered into two types: tissue-specific and constitutional. The qPCR results showed that the expression pattern of the miR156-targeted *NtSPL* gene was tissue-specific, suggesting that miR156-*NtSPL* plays an important role in tobacco growth and development. Cd stress could significantly induce the expression of miR156, and the expression pattern of *NtSPL4a* showed an obvious opposite trend to that of miR156, suggesting that miR156-*NtSPL4a* might mediate the tobacco response to Cd stress. This study lays a foundation for further study of *NtSPL* gene function and provides new insights into the involvement of the *NtSPL* genes in the plant response to heavy metal stress.

## Data Availability

Data are contained within the article and Supplementary Materials.

## Supplementary Materials

The following supporting information can be downloaded at https://www.mdpi.com/article/10.3390/genes14010183/s1: Figure S1: Tobacco Nta-miR156 multiple sequence alignment; Figure S2: Targeting relationship of Nta-miR156 and *NtSPL*; Figure S3: Heatmap of *NtSPL* gene family expression in different tissues of tobacco; Table S1: Tobacco *NtSPL* and Nta-miR156 primers; Table S2: Table of physicochemical properties of the *NtSPL* gene family in tobacco; Table S3: Detailed information of the *cis*-acting elements in the promoters of *NtSPL*.
