# Supplementary material for: The NtSPL Gene Family in Nicotiana tabacum: Genome-Wide Investigation and Expression Analysis in Response to Cadmium Stress
Source: Genes (Basel). 2023 Jan 10;14(1):183. doi: 10.3390/genes14010183 (PMC9859093; doi:10.3390/genes14010183)
Supplement: Supplementary file 1 [file genes-14-00183-s001.zip › Supplementary table 1. Primers list.docx]

Table S1. Primers list

| Primer name | Primer sequences (5’-3’) | Description |
| --- | --- | --- |
| qF-*NtEF1α* | TGAGATGCACCACGAAGCTC | qF-NtEF1α qPCR forward primer |
| qR-*NtEF1α* | CCAACATTGTCACCAGGAAGTG | qR-NtEF1α qPCR reverse primer |
| qF-*NtSPL2a* | GCAGCAGGTGATTCTTGAGAA | qF-NtSPL2a qPCR forward primer |
| qR-*NtSPL2a* | TCAGCTCAATCCATTGAAATAGAAGT | qR-*NtSPL2a* qPCR reverse primer |
| qF-*NtSPL3a* | GAGTTGCAGGAGACGTTTGG | qF-*NtSPL3a* qPCR forward primer |
| qR-*NtSPL3a* | TTCTCTGCCATTGGACTGATG | qR-*NtSPL3a* qPCR reverse primer |
| qF-*NtSPL4a* | AGCCATAATAGGGACAGACACT | qF-*NtSPL4a* qPCR forward primer |
| qR-*NtSPL4a* | GAGAGCAAGCATATCGTATCCA | qR-*NtSPL4a* qPCR reverse primer |
| qF-*NtSPL6a* | CATTCTGACCAAGCGAAGCA | qF-*NtSPL6a* qPCR forward primer |
| qR-*NtSPL6a* | TCTTGTATCGGCTGAATCTCCT | qR-*NtSPL6a* qPCR reverse primer |
| qF-*NtSPL10a* | GCTAATAGTCATGCTGGTGGTA | qF-*NtSPL10a* qPCR forward primer |
| qR-*NtSPL10a* | ATCAGATGTTGGCTTCAGTTCA | qR-*NtSPL10a* qPCR reverse primer |
| qF-*NtSPL13a* | CTACAAGTGGTGGCTGTCAG | qF-*NtSPL13a* qPCR forward primer |
| qR-*NtSPL13a* | CGGAATAACATTTGCTTGGAAG | qR-*NtSPL13a* qPCR reverse primer |
| qF-*NtSPL15a* | TGAAGCTAGCAGCAGTTTGCA | qF-*NtSPL15a* qPCR forward primer |
| qR-*NtSPL15a* | CGTCCAGTGCACATTCTGAACA | qR-*NtSPL15a* qPCR reverse primer |
| qF-*NtSPL17a* | CCAATGATGCTGCGGGAAAT | qF-*NtSPL17a* qPCR forward primer |
| qR-*NtSPL17a* | CCAATGTTCTGGACATCCTTCT | qR-*NtSPL17a* qPCR reverse primer |
| RT-U6 | GTGCAGGGTCCGAGGTTTTGGACCATTTCTCGAT | RT-U6 stem loop primer |
| qF-U6 | GGAACGATACAGAGAAGATTAGCA | qF-U6 qPCR forward primer |
| qF-miR156 | GGACCTGACAGAAGAGAGT | qF-miR156 qPCR forward primer |
| qR-miRNA | GTGCAGGGTCCGAGGT | qR-miRNA qPCR reverse primer |
| RT-miR156 | GTCGTATCCAGTGCAGGGTCCGAGGTATTCGCACTGGATACGACGTGCTC | RT-miR156 stem loop primer |
