# Supplementary material for: The NtSPL Gene Family in Nicotiana tabacum: Genome-Wide Investigation and Expression Analysis in Response to Cadmium Stress
Source: Genes (Basel). 2023 Jan 10;14(1):183. doi: 10.3390/genes14010183 (PMC9859093; doi:10.3390/genes14010183)
Supplement: Supplementary file 1 [file genes-14-00183-s001.zip › Supplementary table 2. Physicochemical properties of the NtSPL family genes.docx]

Supplementary table 2. Physicochemical properties of the *NtSPL* family genes

| Gene Name | Gene ID | mRNA ID | Amino acid size | MW(kDa) | pI | Subcellular localization prediction |
| --- | --- | --- | --- | --- | --- | --- |
| *NtSPL2a* | gene_1596 | XM_016649213.1 | 474 | 51.75 | 8.43 | Nucleus. Cytoplasm |
| *NtSPL2b* | gene_45817 | XM_016602799.1 | 225 | 24.08 | 6.88 | Nucleus |
| *NtSPL2c* | gene_67225 | XM_016629850.1 | 383 | 43.53 | 8.60 | Nucleus. Cytoplasm |
| *NtSPL2d* | gene_32097 | XM_016585575.1 | 360 | 40.26 | 8.61 | Nucleus |
| *NtSPL3a* | gene_26275 | XM_016578265.1 | 140 | 16.00 | 7.00 | Nucleus |
| *NtSPL3b* | gene_46346 | XM_016603464.1 | 136 | 15.64 | 8.18 | Nucleus |
| *NtSPL3c* | gene_72252 | XM_016636254.1 | 133 | 15.27 | 8.81 | Nucleus |
| *NtSPL3d* | gene_64338 | XM_016626070.1 | 136 | 15.79 | 7.67 | Nucleus |
| *NtSPL4a* | gene_52599 | XM_016611390.1 | 171 | 19.32 | 9.33 | Nucleus |
| *NtSPL4b* | gene_69633 | XM_016632969.1 | 167 | 19.00 | 9.36 | Nucleus |
| *NtSPL4c* | gene_49007 | XM_016606866.1 | 181 | 20.62 | 9.65 | Nucleus |
| *NtSPL4d* | gene_2812 | XM_016580674.1 | 182 | 20.63 | 9.63 | Nucleus |
| *NtSPL4e* | gene_64625 | XM_016626454.1 | 165 | 19.24 | 9.13 | Nucleus |
| *NtSPL4f* | gene_24208 | XM_016659701.1 | 172 | 20.22 | 8.66 | Nucleus |
| *NtSPL4g* | gene_32492 | XM_016586100.1 | 119 | 13.66 | 8.83 | Nucleus |
| *NtSPL6a* | gene_20693 | XM_016655244.1 | 524 | 59.47 | 6.80 | Nucleus |
| *NtSPL6b* | gene_42289 | XM_016598217.1 | 523 | 58.20 | 8.36 | Nucleus |
| *NtSPL6c* | gene_14142 | XM_016647076.1 | 508 | 56.63 | 8.36 | Nucleus |
| *NtSPL6d* | gene_19674 | XM_016653897.1 | 494 | 54.46 | 8.52 | Nucleus |
| *NtSPL6e* | gene_8594 | XM_016640008.1 | 524 | 57.20 | 8.97 | Nucleus |
| *NtSPL6f* | gene_35855 | XM_016590259.1 | 522 | 56.91 | 9.04 | Nucleus |
| *NtSPL6g* | gene_52836 | XM_016611687.1 | 542 | 59.66 | 8.52 | Nucleus |
| *NtSPL6h* | gene_39325 | XM_016594405.1 | 538 | 58.90 | 8.45 | Nucleus |
| *NtSPL7a* | gene_29298 | XM_016582110.1 | 389 | 43.49 | 5.29 | Nucleus. Cytoplasm |
| *NtSPL7b* | gene_23677 | XM_016659058.1 | 351 | 38.90 | 5.09 | Nucleus. Cytoplasm |
| *NtSPL8a* | gene_29365 | XM_016582191.1 | 308 | 34.80 | 8.81 | Nucleus |
| *NtSPL8b* | gene_67135 | XM_016629747.1 | 246 | 28.33 | 8.94 | Nucleus |
| *NtSPL8c* | gene_1120 | XM_016643078.1 | 312 | 35.25 | 8.81 | Nucleus |
| *NtSPL10a* | gene_48694 | XM_016606428.1 | 463 | 50.21 | 8.56 | Nucleus. Cytoplasm |
| *NtSPL10b* | gene_17476 | XM_016651175.1 | 462 | 50.17 | 8.19 | Nucleus. Cytoplasm |
| *NtSPL12a* | gene_30740 | XM_016583843.1 | 1000 | 110.87 | 7.28 | Nucleus |
| *NtSPL12b* | gene_22395 | XM_016657360.1 | 1001 | 111.31 | 7.80 | Nucleus |
| *NtSPL12c* | gene_18314 | XM_016652207.1 | 967 | 108.61 | 6.10 | Nucleus |
| *NtSPL12d* | gene_52640 | XM_016611433.1 | 960 | 107.36 | 5.97 | Nucleus |
| *NtSPL12e* | gene_58661 | XM_016619022.1 | 998 | 110.90 | 5.78 | Nucleus |
| *NtSPL12f* | gene_28790 | XM_016581464.1 | 999 | 110.97 | 5.75 | Nucleus |
| *NtSPL13a* | gene_34038 | XM_016588077.1 | 326 | 35.63 | 8.68 | Nucleus |
| *NtSPL13b* | gene_64984 | XM_016626966.1 | 329 | 35.91 | 8.56 | Nucleus |
| *NtSPL15a* | gene_7073 | XM_016634436.1 | 369 | 39.78 | 8.41 | Nucleus |
| *NtSPL15b* | gene_16371 | XM_016649820.1 | 366 | 39.34 | 8.05 | Nucleus |
| *NtSPL17a* | gene_60299 | XM_016621046.1 | 381 | 43.30 | 8.55 | Nucleus |
| *NtSPL17b* | gene_67940 | XM_016630732.1 | 402 | 45.09 | 7.97 | Nucleus |
