# Supplementary material for: The NtSPL Gene Family in Nicotiana tabacum: Genome-Wide Investigation and Expression Analysis in Response to Cadmium Stress
Source: Genes (Basel). 2023 Jan 10;14(1):183. doi: 10.3390/genes14010183 (PMC9859093; doi:10.3390/genes14010183)
Supplement: Supplementary file 1 [file genes-14-00183-s001.zip › Supplementary table 3. Detailed information of the cis-acting elements in the promoters of NtSPL.docx]

|  | **Element** | **Core sequence** | **Function annotation** |
| --- | --- | --- | --- |
| **Plant growth and**  **development** | CAT-box | GCCACT | *cis-*acting regulatory element related to meristem expression |
|  | Circadian | CAAAGATATC | *cis-*acting regulatory element involved in circadian control |
|  | HD-Zip 1 | CAAT(A/T)ATTG | element involved in differentiation of the palisade mesophyll cells |
| **Phytohormone**  **responsiveness** | GARE-motif | TCTGTTG | gibberellin-responsive element |
|  | TCA-element | CCATCTTTTT | *cis-*acting element involved in salicylic acid responsiveness |
|  | ABRE | ACGTG | *cis-*acting element involved in the abscisic acid responsiveness |
|  | TGACG-motif | TGACG | *cis-*acting regulatory element involved in the MeJA-responsiveness |
|  | P-box | CCTTTTG | gibberellin-responsive element |
|  | TATC-box | TATCCCA | *cis-*acting element involved in gibberellin-responsiveness |
| **Stress**  **responsiveness** | ARE | AAACCA | *cis-*acting regulatory element essential for the anaerobic induction |
|  | LTR | CCGAAA | *cis-*acting element involved in low-temperature responsiveness |
|  | TC-rich repeats | GTTTTCTTAC | *cis-*acting element involved in defense and stress responsiveness |
|  | MBS | CAACTG | MYB binding site involved in drought-inducibility |
|  | Wun-motif | AAATTTCCT | wound-responsive element |

Supplementary table 3. Detailed information of the *cis*-acting elements in the promoters of *NtSPL*
