# Supplementary figures and images for: The NtSPL Gene Family in Nicotiana tabacum: Genome-Wide Investigation and Expression Analysis in Response to Cadmium Stress
Source: Genes (Basel). 2023 Jan 10;14(1):183. doi: 10.3390/genes14010183 (PMC9859093; doi:10.3390/genes14010183)

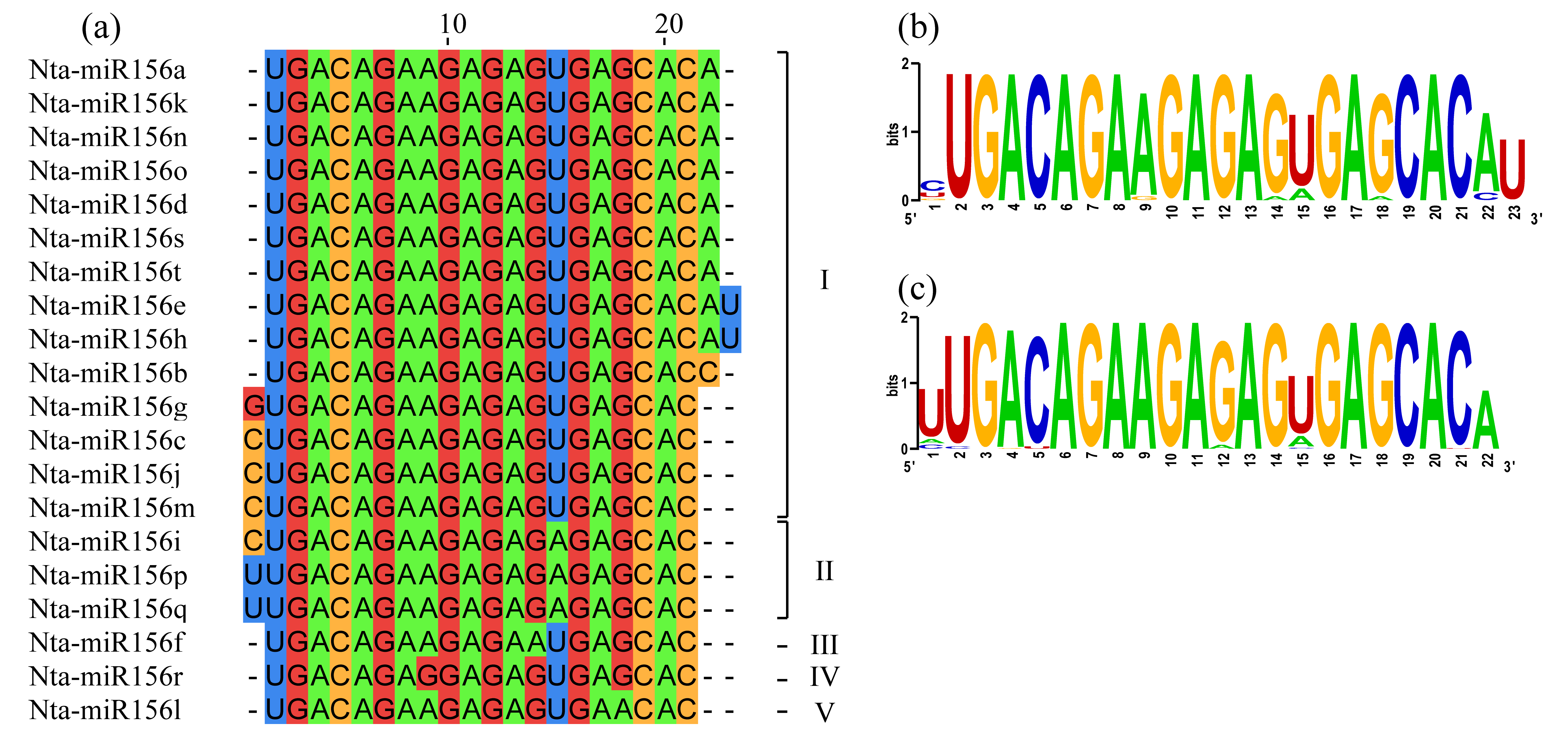

Supplement: Supplementary file 1 [file genes-14-00183-s001.zip › Figure S1. Tobacco Nta-miR156 Multiple Sequence Alignment.tif]

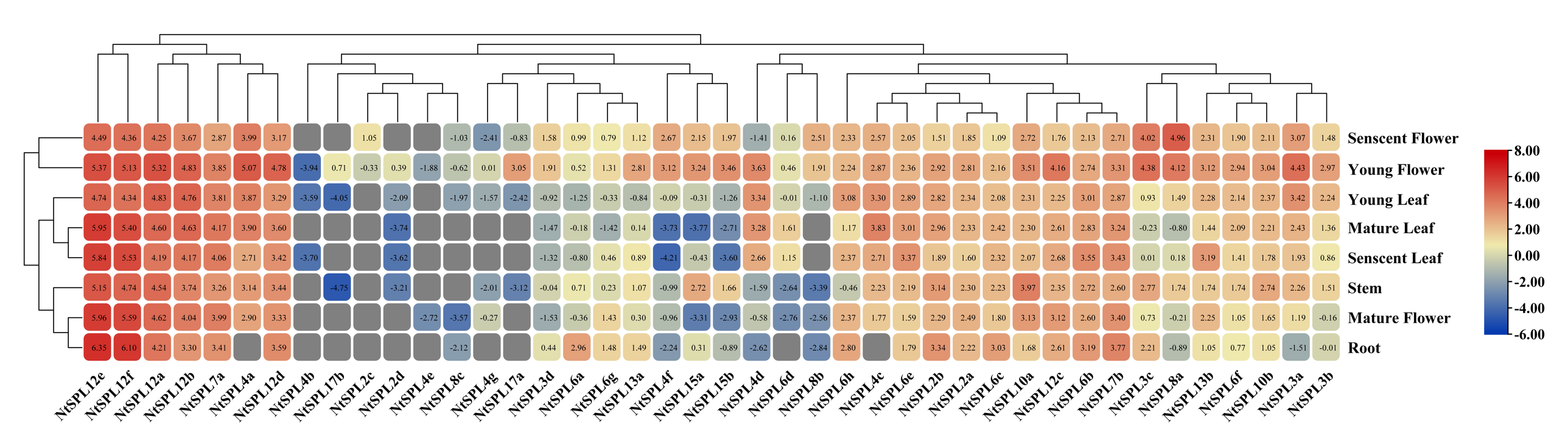

Supplement: Supplementary file 1 [file genes-14-00183-s001.zip › Figure S3. Heat map of NtSPL gene family expression in different tissues of tobacco.tif]
